# A modified Delphi study to gain consensus for a taxonomy to report and classify physical activity referral schemes (PARS)

**DOI:** 10.1186/s12966-020-01050-2

**Published:** 2020-12-02

**Authors:** Coral L. Hanson, Emily J. Oliver, Caroline J. Dodd-Reynolds, Alice Pearsons, Paul Kelly

**Affiliations:** 1 School of Health and Social Care, Edinburgh Napier University, Sighthill Campus, Edinburgh, EH11 4DN UK; 2 Department of Sport and Exercise Sciences, Durham University, Durham, DH1 3HN UK; 3 Physical Activity for Health Research Centre, Institute for Sport, Physical Education and Health Sciences, University of Edinburgh, Edinburgh, EH8 8AQ UK

**Keywords:** Physical activity, Public health, Exercise referral, Evaluation, Rehabilitation medicine

## Abstract

**Background:**

Physical Activity Referral Schemes (PARS), including exercise referral schemes, are a popular approach to health improvement, but understanding of effectiveness is limited by considerable heterogeneity in reporting and evaluation. We aimed to gain consensus for a PARS taxonomy as a comprehensive method for reporting and recording of such schemes.

**Methods:**

We invited 62 experts from PARS policy, research and practice to complete a modified Delphi study. In round one, participants rated the need for a PARS taxonomy, the suitability of three proposed classification levels and commented on proposed elements. In round two, participants rated proposed taxonomy elements on an 11-point Likert scale. Elements scoring a median of ≥7, indicating high agreement, were included in the final taxonomy.

**Results:**

Of those invited, 47 (75.8%) participated in round one, with high retention in round two (*n* = 43; 91.5%). 42 were UK-based, meaning the resultant taxonomy has been scrutinised for fit to the UK context only. The study gained consensus for a three-level taxonomy: Level 1: PARS classification (primary classification, provider, setting, conditions accepted [have or at risk of], activity type and funding). Level 2: scheme characteristics (staff structure, staff qualifications, behaviour change theories, behaviour change techniques, referral source, referrers, referral process, scheme duration, session frequency, session length, session times, session type, exit routes, action in case of non-attendance, baseline assessment, exit assessment, feedback to referrer and exclusion criteria) and Level 3: participant measures (demographics, monitoring and evaluation, and measures of change).

**Conclusion:**

Using a modified Delphi method, this study developed UK-based consensus on a PARS classification taxonomy. We encourage PARS practitioners and public health colleagues, especially those working with similar service models internationally, to test, refine and use this taxonomy to inform policy and practice.

**Supplementary Information:**

The online version contains supplementary material available at 10.1186/s12966-020-01050-2.

## Introduction

Physical inactivity is responsible for 6.4% of global mortality, contributing an estimated $53.8 billion to healthcare costs worldwide in 2013 [1]. Adults who are more active have better physical and mental health, and higher quality of life [2]. Despite this, globally, more than one in four people were insufficiently active in 2018, with women less active than men [2].

Exercise referral schemes, established in the 1990’s, are an internationally recognised way to ‘prescribe’ activity to enable people to achieve recommended levels of PA [3, 4]. In the UK, traditionally, a patient with a health condition, or other factors putting them at risk of ill health, would be referred by a healthcare professional if they were sedentary or inactive. This was followed by referral to a PA specialist/service, a personal needs assessment and an opportunity to participate in PA over 10 weeks or longer [5–7]. Other nations have exercise referral systems, however variation exists in terms of policies, referral mechanisms and practices. In some countries healthcare professionals ‘prescribe’ exercise rather than fitness professionals, although PA usually takes place in the community as it does in the UK. For example, Sweden’s ‘Physical Activity on Prescription’ model, currently being trialled in other European countries, [8] consists of healthcare professionals having a patient-centred discussion and producing an individually tailored written PA prescription, which is followed-up by the same prescriber [9]. Similarly, the American College of Sports Medicine’s ‘Exercise is Medicine’ model stresses PA brief advice and basic exercise prescription by healthcare professionals after an assessment of readiness to change PA behaviour. Mechanisms for establishing referrals to fitness professionals do exist, [10] making some aspects of Exercise is Medicine comparable to UK schemes. The Australian model is the most similar to the UK model, as it allows referrals specifically to exercise physiologists where a chronic medical condition has been diagnosed [11].

In the UK, physical activity services have broadened in recent years and (as we have previously reported) the term ‘exercise referral’ now fails to accurately represent the range of schemes available [12]. This is the case both in terms of evidence-informed models and contemporary practices, such as social prescribing, where patients can be referred via link workers into local community groups (across health, arts, culture and sport) and self-referral, where an individual can request direct entry into a scheme, rather than referral by a healthcare practitioner. We have suggested that the broader and more inclusive definition of ‘physical activity referral schemes’ (PARS), which includes all schemes that offer supported PA options/choices for individuals with a health condition, recognises such recent innovation in supporting PA uptake [12].

Current understanding of PARS effectiveness is limited by considerable heterogeneity in available data reported both by individual schemes [13, 14] and at systematic review level [15, 16]. Quality of reporting is variable: e.g. around scheme delivery components and processes or evaluation-based appraisals [17]. The result is ambiguity in understanding of effectiveness and a lack of policy guidance for best practice or practices (e.g. if best practice varies by context or client type), and a lack of understanding about 'what good looks like' [17]. A shift in recognition and understanding of PARS is needed to advance policy, evidence and practice. There is a clear need for full documentation of practical details within scheme provision and delivery in order to understand the existing heterogeneity across schemes nationally and internationally. This will better-inform future policy, commissioning, and delivery.

We therefore propose the generation of a comprehensive framework for reporting and recording scheme details. To achieve this, a rigorous process must be undertaken to extract critical knowledge and insight from PARS experts across policy, research and practice. A Delphi study is a widely adopted and well-accepted systematic method to achieve consensus of expert opinion [18, 19]. We report the process and findings from a modified Delphi study, undertaken to inform the development of a PARS classification taxonomy, grounded in both theory and practice-based experience.

## Method

**Fig. 1:**
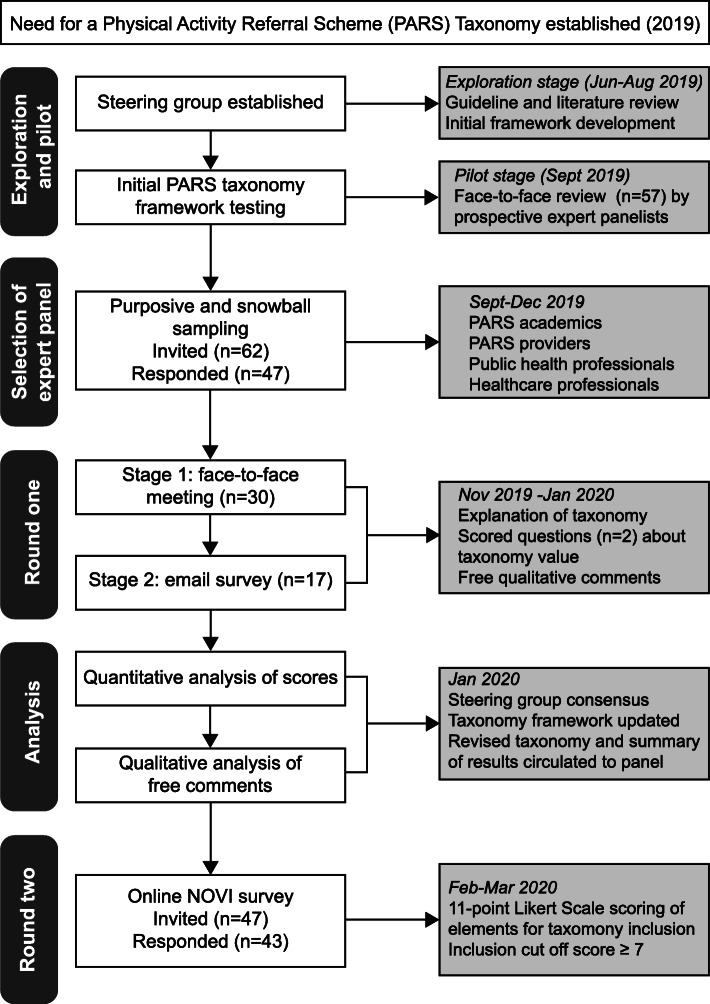
Flow diagram of modified Delphi study process

A modified Delphi study that gained UK-based consensus for a PARS taxonomy (Fig. 1) following the guidance on Conducting and REporting of DElphi Studies (CREDES) [20].

### Exploration and pilot stage

We established a steering group of four UK-based researchers with expertise in PARS, behaviour change theory and research/evaluation methods (CH, PK, CDR, EO) to develop an initial taxonomy framework. A preliminary review of relevant UK guidelines, literature and steering group perspectives informed the development of a prototype three-level PARS taxonomy. To allow for the gathering of wider viewpoints, two researchers (CH, PK) presented the prototype taxonomy to PARS and public health experts (*n* = 57) at a Scottish NHS Information Exchange in September 2019. After the presentation, we encouraged open-ended discussion and made field notes about comments. We asked attendees to provide written comments on a printed version of the taxonomy, and promoted both supportive and conflicting feedback. The author team considered all observations and updated the framework accordingly. The proposed taxonomy consisted of a high-level classification figure and a checklist of PARS elements [12].

### Recruitment of expert panel

We defined experts as ‘individuals involved in the conception, design, conduct, teaching or analysis of PARS.’ We used purposive and snowball sampling to invite more than the suggested minimum 20 contributors [21] to form an expert panel. Professional backgrounds included (a) academics/researchers and published authors in the PARS field, (b) providers of PARS and (c) public health and healthcare professionals involved in PARS. For invited participants, there were no exclusion criteria. We invited experts to participate via email and provided participant information packs. We received expressions of interest from people in five different countries (UK, Ethiopia, Canada, Ireland and Australia), but only one participant from outside the UK (Australia) completed the study, making the results valid for UK-based PARS. To reduce the risk of bias from the steering group, an independent researcher (AP) co-ordinated the study.

### Round one

We conducted an initial face-to-face meeting in November 2019 as part of a free one-day specialist workshop, with invited academic presentations from UK PARS researchers. We advertised the workshop via UK national public health networks, and international university networks and social media. We followed this with a second stage of remote email questionnaires for those unable to attend the meeting or who contacted us via snowball sampling. All participants gave written signed consent. At the face-to-face meeting, we formally presented the taxonomy before asking panellists to complete the first survey round. For email panellists we provided an explanation document. During the meeting, we used Slido (https://www.sli.do/) to collect anonymous feedback and asked experts to score two introductory questions on an 11-point Likert scale (0-Do not agree at all, 10-Agree very strongly):

1. Do you believe there is value in developing a PARS taxonomy?
2. Do you agree that we need a three-level approach (primary classification, scheme characteristics and participant measures) to the proposed taxonomy?

We then asked panellists to move systematically through the taxonomy and make free comments about each element. We encouraged both supportive and critical feedback, and requested detailed justification for any suggestions of missed or superfluous items to help revise the next iteration of the taxonomy. The steering group analysed quantitative scoring, and collated and discussed qualitative comments, before adapting the proposed taxonomy prior to the second round.

### Round two

In round two, we disseminated an updated taxonomy, an explanation of changes and a summary of anonymised first round comments to the expert panel. We asked panellists to complete an online NOVI survey (https://novisurvey.net/) and score each taxonomy element on an 11-point Likert scale (0-Unimportant, do not include in taxonomy, 10-Very important, must be included in taxonomy). Using a measure of central tendency as a definition of consensus, [22] we informed participants that the cut off for element inclusion in the taxonomy was a score of ≥7. We asked panellists to comment on, but not score, sub-elements (e.g. conditions accepted by schemes). This was because the taxonomy design allowed for local adaptations (e.g. the addition of a further medical condition, while maintaining conditions identified earlier in the process as being most common), thus did not cover all potential variations of scheme provision.

### Data analysis

Quantitative data were analysed using SPSSv26 (IBM, NY, USA). We examined the data distribution of each score by calculating z-scores for skewness and kurtosis, and via Shapiro-Wilks tests. We reported the median and interquartile range (IQR) of overall scores for each taxonomy element and by professional group. Items scoring ≥7 overall were included in the final taxonomy.

Our approach to responding to comments raised through the consultation phases adopted three basic principles. (1) We reviewed all comments taking into account the number of panellists making similar comments. We acted on comments made by one individual in exception, as these were considered outlying discrepant viewpoints from the consensus. (2) Where we considered that suggestions for additions to any taxonomy element could be adequately recorded in an ‘other’ category, we did not make an amendment. (3) All authors discussed responses to comments and made decisions about subsequent actions as a team. We agreed small changes to sub-elements provided we could sufficiently justify them based on qualitative comments and author group consensus.

## Results

In total, we invited 62 experts to participate (inviting 36 to a face-to-face meeting and 26 via snowball sampling and social media advertising). We received 47 responses (75.8% response rate). Thirty (63.8%) attended the face-to-face meeting and 17 (36.2%) responded via an email questionnaire. We only invited respondents from round one to participate in round two. Forty-three (91.5% of round one respondents) completed round two. Of the 43 study completers, 16 (37.2%) were academics/researchers, 12 (27.9%) were PAR providers/commissioners, six (14.0%) were both a researcher and a PARS provider, and nine (20.9%) were public health/healthcare professionals. Most panellists were UK-based, with one from Australia, validating the taxonomy in a UK context.

### Round one results

Participants considered there was value in developing a PARS taxonomy (median score 10.0, [IQR 9.0–10.0]) and that a three-level approach (primary classification, scheme characteristics and participant measures) was appropriate (median score 8.0, [IQR 7.0–9.5]).

Based on qualitative comments from panellists, we made minor amendments to most elements of the initial taxonomy. We made five major changes. First, in the figure, we redefined level 1a (primary classification) to better reflect types of schemes offered, removing the previous terminology of ‘traditional’ versus ‘non-traditional’ PARS.

We received some comments that PARS must include explicit behaviour change support but not all panellists agreed, stating that schemes without explicit behaviour change support should be included in the taxonomy. Second, we moved funding (level 1e) up into the PARS classification level of the taxonomy from level 2, reflecting its importance in determining scheme type.

Third, in the checklist, we changed level 1a to include two sets of questions that would allow for high-level scheme classification (Table 1).

**Table 1:** Level 1a: primary classification questions

**Physical Activity Referral Scheme (PARS) Reporting Checklist**	
**Level 1 PARS classification**	
**Level 1a: Primary classification** The purpose of this taxonomy is to provide a classification system for PARS, including clinically based exercise schemes, exercise referral schemes and social prescribing for physical activity (PA). It is for use in evidence reviews of delivery and effectiveness. It is also an audit and monitoring tool for funders and providers to capture service delivery. The taxonomy is intended for programmes that fulfil **all of the following three criteria**:	Tick all that apply
1. Have a primary aim of increasing PA	
2. Have a formalised referral process^a^	
3. Are for individuals who are inactive and/or sedentary, and/or have (*or are at risk of having)* a health condition	
**If you have not ticked all of these boxes, then the PARS taxonomy is not suitable for your programme.**	
Additionally programmes **may** also include the following	Tick any that apply
1. Individual behaviour change consultations	
2. PARS specialist staff supervised PA sessions or one-to-one supervision	
3. Signposting to a range of available activities	

^a^ An agreed and documented process for the transfer of referral information between health/social care referrers and PARS providers, which leads to individuals being able to access PARS

Fourth, we amended level 2c (behaviour change theories) to a more general question about whether a PARS was based on behaviour change theory, and added a further section (level 2d, behaviour change techniques) as these were considered more easily identifiable by providers completing the taxonomy. Finally, we added in three new subsections to scheme characteristics (level 2j, session length, level 2k, session times and level 2s, exclusion criteria). We made minor changes to the details within taxonomy subsections, but recognised that these could not reflect every variation of every scheme, so where appropriate ensured each subsection had an ‘other’ element.

### Round two results

#### Scoring of elements for inclusion in the PARS taxonomy

The scores for each element did not follow a normal distribution. With the exception of ‘equipment loan’, all taxonomy elements scored a median of ≥7 and were included in the final consensus (Table 2). The median score for PARS providers was below ≥7 for length and time of sessions, but overall these items gained consensus.

**Table 2:** Round two scoring of elements for inclusion in the PARS taxonomy

Taxonomy element	Academic/Researcher (*n* = 16)		PARS provider/ commissioner (*n* = 12)		Both researcher and PARS provider (*n* = 6)		Public health or healthcare professional (*n* = 9)		All panellists (*n* = 43)	
	Median	IQR	Median	IQR	Median	IQR	Median	IQR	Median	IQR
**LEVEL 1 PARS classification**										
1a) Primary classification	9.5	8.3–10.0	9.5	8.0–10.0	10.0	9.8–10.0	10.0	6.0–10.0	**10.0**	8.0–10.0
1b) Provider	10.0	9.0–10.0	8.0	4.8–9.8	10.0	8.8–10.0	9.0	7.5–9.5	**9.0**	8.0–10.0
1b) Setting	10.0	9.0–10.0	7.0	7.0–8.0	10.0	8.8–10.0	9.0	7.5–9.5	**9.0**	7.0–10.0
1c) Conditions accepted (have or at risk of)	10.0	9.3–10.0	10.0	8.3–10.0	10.0	8.8–10.0	10.0	8.0–10.0	**10.0**	9.0–10.0
1d) Activity type	10.0	9.3–10.0	9.5	7.0–10.0	9.0	6.5–10.0	9.0	8.0–9.5	**10.0**	8.0–10.0
1e) Funding	10.0	8.0–10.0	7.0	5.0–8.8	8.0	6.5–9.3	9.0	5.5–10.0	**8.0**	7.0–10.0
**LEVEL 2 Scheme characteristics**										
2a) Staff structure	9.0	7.3–10.0	8.5	3.5–9.8	9.5	5.8–10.0	8.0	4.5–8.5	**9.0**	6.0–10.0
2b) Staff qualifications	10.0	9.0–10.0	10.0	9.0–10.0	10.0	9.0–10.0	9.0	7.0–10.0	**10.0**	9.0–10.0
2c) Behaviour change theories	9.0	6.3–10.0	7.0	6.3–9.5	10.0	8.8–10.0	8.0	5.5–10.0	**8.0**	7.0–10.0
2d) Behavioural change techniques	9.0	8.0–10.0	8.0	7.0–10.0	10.0	8.8–10.0	8.0	5.0–10.0	**9.0**	7.0–10.0
2e) Referral source	9.0	7.0–10.0	8.0	7.0–10.0	9.5	7.8–10.0	9.0	7.5–10.0	**9.0**	7.0–10.0
2f) Referrers	10.0	9.3–10.0	8.0	7.0–9.8	9.5	7.5–10.0	9.0	6.5–10.0	**9.0**	8.0–10.0
2g) Referral process	9.5	6.5–10.0	8.0	7.0–10.0	10.0	8.5–10.0	8.0	4.5–10.0	**9.0**	7.0–10.0
2h) Scheme duration	10.0	10.0–10.0	9.0	7.0–10.0	10.0	9.0–10.0	10.0	8.0–10.0	**10.0**	9.0–10.0
2i) Session frequency	10.0	10.0–10.0	9.0	5.5–10.0	9.5	7.3–10.0	9.0	8.0–10.0	**10.0**	8.0–10.0
2j) Session length	10.0	7.5–10.0	6.5	5.0–7.8	9.5	5.3–10.0	9.0	7.5–9.5	**9.0**	6.0–10.0
2k) Session time	8.0	5.0–10.0	6.0	2.0–7.0	7.0	5.3–10.0	8.0	6.5–9.0	**7.0**	5.0–9.0
2l) Session type	10.0	9.3–10.0	8.0	7.0–10.0	10.0	8.5–10.0	9.0	7.5–10.0	**10.0**	8.0–10.0
2m) Equipment loan^a^	5.0	3.3–7.8	0.5	0.0–5.0	6.0	4.5–7.0	5.0	4.0–7.0	**5.0**^a^	3.0–7.0
2n) Exit routes	9.5	9.0–10.0	9.0	7.3–10.0	9.5	7.0–10.0	8.0	7.5–10.0	**9.0**	8.0–10.0
2o) Action in case of non-attendance	9.5	9.0–10.0	8.5	4.3–10.0	10.0	7.5–10.0	8.0	7.5–10.0	**9.0**	8.0–10.0
2p) Baseline assessment	10.0	10.0–10.0	10.0	9.3–10.0	10.0	9.0–10.0	10.0	7.5–10.0	**10.0**	10.0–10.0
2q) Exit assessment	10.0	10.0–10.0	10.0	10.0–10.0	10.0	9.0–10.0	10.0	7.5–10.0	**10.0**	10.0–10.0
2r) Feedback provided to referrer	10.0	9.0–10.0	7.0	5.0–9.8	9.0	7.0–10.0	8.0	7.5–9.5	**9.0**	7.0–10.0
2s) Exclusion criteria	10.0	9.3–10.0	10.0	7.3–10.0	9.5	7.8–10.0	10.0	8.5–10.0	**10.0**	9.0–10.0
**LEVEL 3 Participant characteristics**										
3a) Demographics	10.0	9.3–10.0	10.0	8.5–10.0	9.5	8.3–10.0	10.0	7.0–10.0	**10.0**	9.0–10.0
3b) Number of referrals	10.0	9.3–10.0	10.0	7.3–10.0	9.5	7.8–10.0	10.0	7.0–10.0	**10.0**	8.0–10.0
3c) Uptake, attendance and adherence	10.0	10.0–10.0	10.0	8.5–10.0	9.5	8.5–10.0	10.0	8.0–10.0	**10.0**	9.0–10.0
3d) Measures of Change	10.0	10.0–10.0	10.0	8.5–10.0	9.5	8.5–10.0	10.0	8.5–10.0	**10.0**	9.0–10.0

^a^Excluded from the final taxonomy

#### Level 1: primary classification

Sixteen panellists commented on primary classification. Overall, they consistently supported the main elements in this taxonomy level and comments were mainly confirmatory. However, panellists suggested clarification of key terms, additional sub-elements, or minor edits to wording. We agreed five minor amendments to sub-elements of level 1b (provider and setting) and 1d (activity type) in both the figure and the checklist. These included adding an option for a clinical setting, since panellists felt that the taxonomy was valuable for clinical exercise programmes. We changed wording to request additional detail for all level 1 elements in the third column of the checklist (e.g. specifying the exact venue and location of PARS setting). Finally, we added detail to level 1a questions in the checklist; *‘explicit, planned behaviour change techniques included e.g. goal setting, formalised activity tracking/activity monitoring’* to clarify the meaning of individual behaviour change consultations and examples of signposting to generic activities ‘*e.g. walking football, Pilates and Zumba’*.

#### Level 2: scheme characteristics

Eighteen panellists commented on scheme characteristics. As with level 1, many comments confirmed agreement with the elements in level 2. Seven minor edits were made to item and checklist wording, including the addition of examples to aid clarity (e.g. of PARS specific qualifications). Additionally, we discussed two other areas of feedback.

First, comments questioned the necessity of retaining both level 2e (referral source) and level 2f (referrers) categories. We noted potential difficulties in categorising primary, secondary or tertiary care referrals based on referrer role title, as some job titles span multiple domains. We considered that the ability to examine schemes based on where (in the healthcare continuum) PARS received referrals from, was important and therefore retained both levels in the final taxonomy. Second, some panellists commented that exclusion criteria listed were too specific, having different terminology between schemes, and focussing overly on medical criteria for exclusion. In response, we decided to remove the list and ask users to detail exact exclusion criteria.

#### Level 3: participant characteristics

Thirteen panellists commented on participant characteristics, reporting that recording demographics was important to create local and national evidence of impact, especially in gauging whether PARS successfully targeted those with the largest health and PA inequalities. Panellists considered some demographics more important (age, gender and socio-economic status). We did not remove any demographic sub-category, however noted a requirement to stress that the taxonomy is not prescriptive, but is a tool to classify schemes: what is being delivered, by whom and how.

One panellist questioned whether elements 3b-3d (number of referrals, uptake, attendance and adherence, and measures of change) were about evaluation rather than the stated taxonomy aim of describing PARS. This panellist suggested that these elements should have a sub-heading of ‘monitoring and evaluation’ above level 3b for clarity. We agreed this was an important suggestion and added the new subheading to create an area where users could record what is, or has been evaluated, and how. However, the checklist does not mandate that users must conduct an evaluation in order to complete the taxonomy.

Some panellists identified repetition between the level 2n/2o categories ‘baseline assessment’ and ‘exit assessment’ and level 3d ‘measures of change’. We removed details of the measures from level 2n/2o. We considered the minor changes made to sub-elements were justifiable posteriori considerations, [18] and that the Delphi study had reached consensus, as all agreed elements were retained in the taxonomy.

**Fig. 2:**
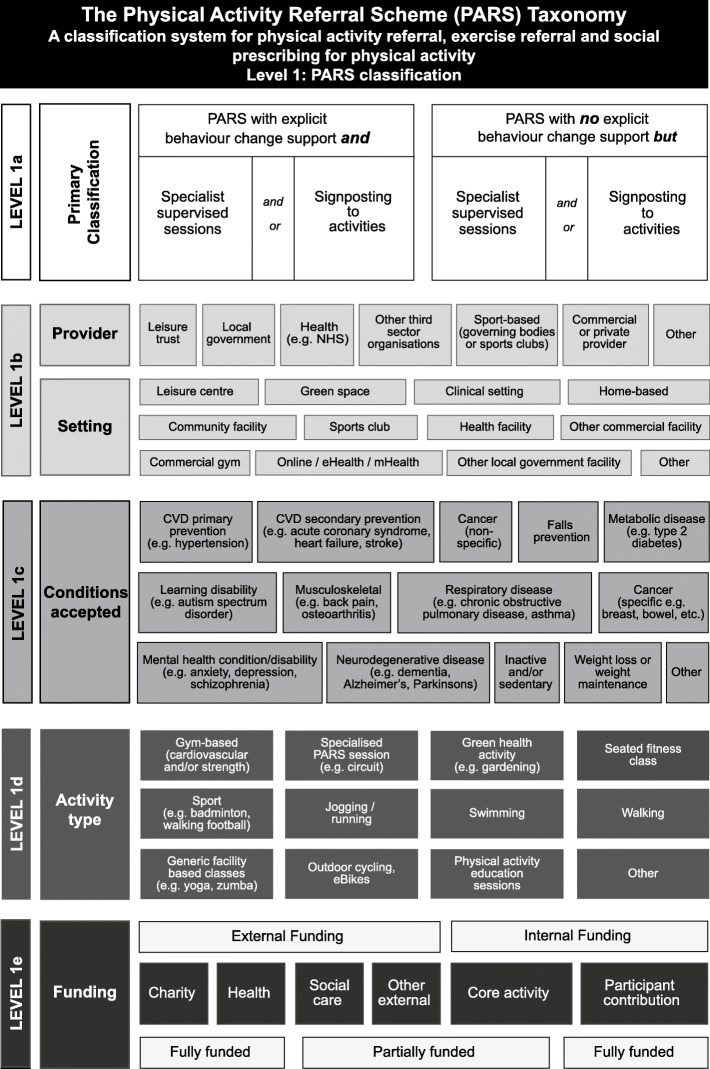
Level 1: PARS classification

**Fig. 3:**
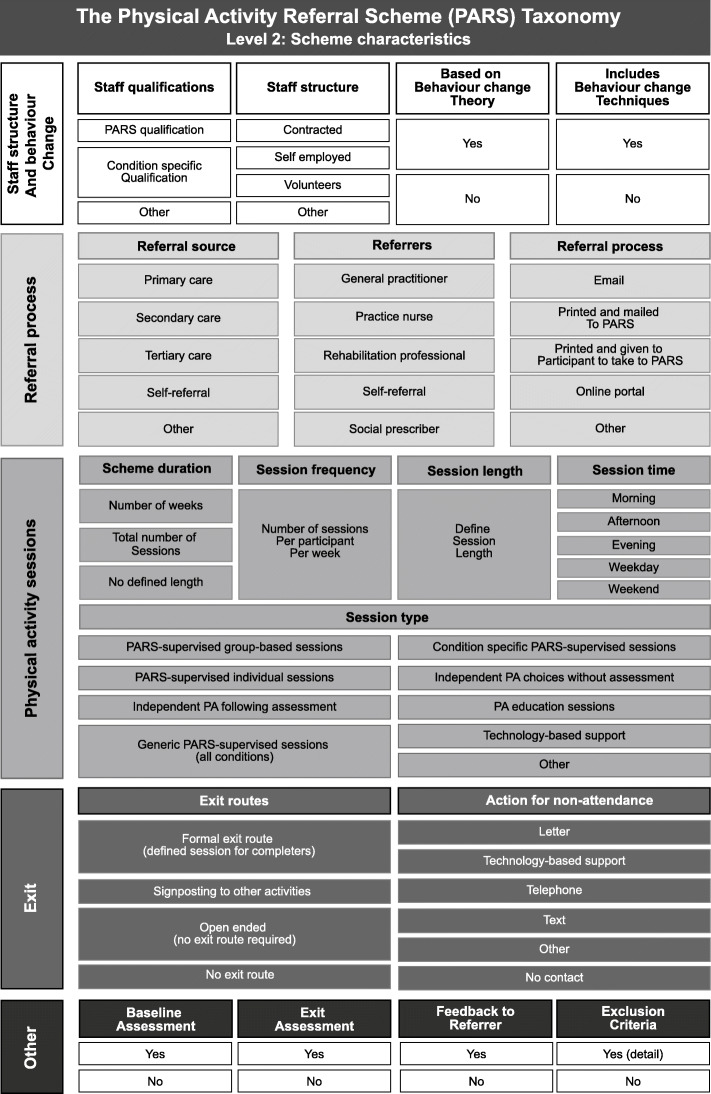
Level 2: Scheme characteristics

**Fig. 4:**
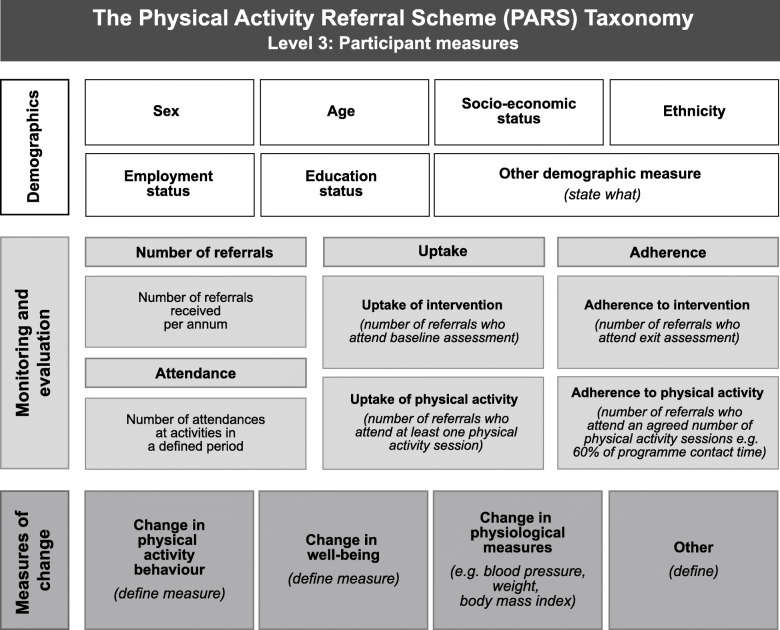
Level 3: Participant measures

The study resulted in the creation of a Level 1: PARS classification diagram (Fig. 2), a Level 2: Scheme characteristics diagram (Fig. 3), a level 3: Participant measures diagram (Fig. 4) and a taxonomy checklist (see Additional file 1).

## Discussion

This research used a modified Delphi method that developed a UK validated PARS classification taxonomy endorsed by academics, practitioners, and scheme providers. In doing so, we have developed an expert-informed tool, perceived as both functionally viable by intended users and informative for stakeholders. Hence, we encourage PARS providers and researchers to report consistently and publicly scheme characteristics using the taxonomy. Doing so will facilitate the generation of robust and comprehensive data to better understand the nature of PARS provision. Given the proliferation in, and differentiation of, PARS practices, we are at a critical point in terms of ensuring that understanding of what works, for whom, can be advanced. Importantly, standardising reporting will enable the collation of data from similar schemes and inform between-scheme comparisons recommended by previous authors [10, 12]. This will facilitate future monitoring and evaluation, essential for appropriately, and effectively supporting public health policy and practice [23].

### The inclusion of behaviour change theory and techniques in PARS

The necessity, or otherwise, of including behaviour change theory and techniques in PARS was the most debated element of this study. Some panellists, generally providers, considered that the ‘traditional’ necessity in the UK [6, 7] of including formal behaviour change led to limited recognition of many contemporary PARS. The evidence for the successful implementation of formal behaviour change into PARS is equivocal, [24, 25] but there has been little examination of whether informal behaviour change techniques are integral to such schemes. Given this, we ensured that the taxonomy captured the presence or absence of individual behaviour change consultations at level 1, and that level 2 provided an opportunity for detailed capture of any underlying theoretical principles and behaviour change techniques included in PARS. Given that behaviour change taxonomies developed elsewhere have, for example close to 100 elements, [26] with up to 21 techniques based on one specific theory, [27] we decided not to include examples. This prevents any risk of biasing or narrowing responses.

### Strengths and limitations

Delphi studies provide a means to arrive at justifiable, valid and credible solutions based on expert judgement [28]. However, we recognise that the decision-making process inevitably involves subjectivity and judgemental inputs, in terms of panel selection, item selection, and resolution of contention [29]. While we ensured anonymity between participants (using anonymous software and online-data capture), not all researchers were always blind to comments when returned via email. Lastly, panel consensus methods can involve a ‘watering down’ of opinions and ideas [30]. It is possible that the taxonomy may need future development to capture particularly innovative or emerging characteristics linked with scheme effectiveness.

We are aware that the Delphi panel were UK-centric, and invite further refinement of the taxonomy from international colleagues, including non-PARS focused experts. Given that models in some other countries focus on healthcare professionals providing an exercise prescription, [9, 10] further consultation with international experts may highlight a need for adaptations to the taxonomy to make it more relevant in these countries.

The challenges of capturing evaluation data and evaluation in health programmes are widely discussed [31]. The PARS taxonomy, formed by expert consensus across a range of UK stakeholders, represents a first step in producing a robust system for capturing detailed programme delivery, structure and operational detail that is required for between-programme evaluation to identify best practice or practices in the UK.

### Implications for policy and practice

We suggest that the PARS taxonomy is used to classify, record and report PARS delivery. It is our intention that this would be done at a “scheme level” so that a provider delivering different PARS would complete separate reporting checklists for each scheme. If providers are delivering a generic scheme for different medical conditions, we suggest that medical condition is recorded at individual participant level to allow for assessment of success for different conditions.

We also suggest a number of recommendations for future development. First, that data generated from use of the taxonomy should be audited regularly to identify required adaptations based on changing provision landscapes. Revisions of the taxonomy should invite stakeholder engagement in the same way as the present version, and be publicly available for use. Second, stakeholders should collaborate to develop systems and processes for data sharing and subsequent evaluation, as well as disseminating findings back to practitioners. This should include national and regional organisations who are using the taxonomy streamlining collection, processing and analysis of data in effective monitoring and evaluation systems. Without linking the taxonomy to accompanying outcome evaluation data, understanding of effective and ineffective practices is constrained. Third, we encourage the user to have flexibility to go to the level of depth appropriate. With minimal time and capacity they should rapidly be able to complete level one to provide a high-level monitoring system with information about scheme type (a minimum dataset so to speak). When a more in depth evaluation is warranted the information at levels 2 and 3 can be collected and analysed as well, but still in harmony with data from PARS that only collect/report at level 1. Lastly, while we accept that local variations may be useful to capture subtle differences in provision, we recommend that the present taxonomy is seen as a minimum reporting standard. While elements may be added to the taxonomy, we advise against removal of elements from the level at which it is being used. Instead, users should note ‘not applicable’ or ‘not available’ (as with monitoring and evaluation above). This is important to maintain the intended purpose of capturing comparable data across schemes.

## Conclusion

This research used a modified Delphi method to develop a UK-based consensus on a PARS classification taxonomy. We suggest that the reporting taxonomy complements and is used alongside UK policy guidance in designing, monitoring and evaluating PARS. In addition, we encourage international consideration and feedback about suitability for use outside the UK and would welcome further collaboration to develop an international consensus for a PARS taxonomy.

## Supplementary Information

**Additional file 1.**

## Data Availability

The datasets used and/or analysed during the current study are available from the corresponding author on reasonable request.
