# Supplementary material for: A modified Delphi study to gain consensus for a taxonomy to report and classify physical activity referral schemes (PARS)
Source: Int J Behav Nutr Phys Act. 2020 Dec 2;17:158. doi: 10.1186/s12966-020-01050-2 (PMC7709269; doi:10.1186/s12966-020-01050-2)
Supplement: Supplementary file 1 — Additional file 1. [file 12966_2020_1050_MOESM1_ESM.docx]

| **Physical Activity Referral Scheme (PARS) Reporting Checklist** | | |
| --- | --- | --- |
| **Level 1 PARS classification** | | |
| **Level 1a: Primary classification**  The purpose of this taxonomy is to provide a classification system for PARS, including clinically based exercise schemes, exercise referral schemes and social prescribing for physical activity (PA). It is for use in evidence reviews of delivery and effectiveness. It is also an audit and monitoring tool for funders and providers to capture service delivery. The taxonomy is intended for programmes that fulfil all of the following **three criteria**: | | Tick all that apply |
| 1. Have a primary aim of increasing PA | |  |
| 1. Have a formalised referral process | |  |
| 1. Are for individuals who are inactive and/or sedentary, and/or have (*or are at risk of having)* a health condition. | |  |
| **If you have not ticked all of these boxes, then the PARS taxonomy is not suitable for your programme.** | | |
| Additionally programmes **may** also include the following | | Tick any that apply |
| 1. Individual behaviour change consultations *(explicit, planned behaviour change techniques included e.g. goal setting, formalised activity tracking/activity monitoring)* | |  |
| 1. PARS specialist staff supervised PA sessions or one-to-one supervision | |  |
| 1. Signposting to a range of generic available activities delivered by non-PARS specialist staff *(e.g. walking football, yoga, Pilates, Zumba)* | |  |
| **1b Provider** | Tick all that apply | Further specify provider |
| Leisure trust |  |  |
| Local government |  |  |
| Other third/community sector organization |  |  |
| Sport-based (governing bodies or sport clubs) |  |  |
| Commercial/private provider |  |  |
| Health (e.g. NHS) |  |  |
| Other (define) |  |  |
| **1b Setting** | Tick all that apply | Specify exact location (e.g. leisure centre name and location, including postcode or web link/app name etc.) |
| Leisure centre |  |  |
| Green/outdoor space (define) |  |  |
| Sports club |  |  |
| Community facility (define) |  |  |
| Commercial gym |  |  |
| Other commercial facility (define) |  |  |
| Other local government facility (define) |  |  |
| Home-based |  |  |
| Clinical setting |  |  |
| Online/eHealth/mHealth |  |  |
| Other (define) |  |  |
| **1c Conditions accepted (have or at risk of)** | Tick all that apply | Specify exact conditions within each subsection |
| Cardiovascular primary prevention (e.g. hypertension) |  |  |
| Cardiovascular secondary prevention (e.g. acute coronary syndrome, heart failure, stroke) |  |  |
| Respiratory disease (e.g. chronic obstructive pulmonary disease, asthma) |  |  |
| Metabolic disease (e.g. type 2 diabetes) |  |  |
| Mental health condition/disability (e.g. anxiety, depression, schizophrenia) |  |  |
| Learning disability (e.g. autism spectrum disorder) |  |  |
| Musculoskeletal (e.g. back pain, osteoarthritis) |  |  |
| Cancer (nonspecific) |  |  |
| Cancer specific (e.g. breast, bowel) |  |  |
| Weight loss or weight maintenance |  |  |
| Falls prevention (primary and secondary prevention) |  |  |
| Neurodegenerative disease (dementia, Alzheimer’s Parkinson’s) |  |  |
| Inactive and/or sedentary |  |  |
| Other (define) |  |  |
| **1d Activity type** | Tick all that apply | Further specify activities |
| Gym-based (cardiovascular and/or strength) |  |  |
| PARS specialized class led by PARS qualified staff (e.g. U.K level 3 exercise referral qualification) |  |  |
| Walking |  |  |
| Jogging/running |  |  |
| Swimming |  |  |
| Outdoor cycling, e-bikes |  |  |
| Sport (e.g. badminton, walking football) |  |  |
| Seated fitness class |  |  |
| Generic fitness class (e.g. yoga, aerobics, Zumba) |  |  |
| Gardening/green gym or other green health activity |  |  |
| PA education sessions |  |  |
| Other (define) |  |  |
| **1e Funding** | Tick all that apply | Please state exact funding source, level of funding per participant and length of funding agreement |
| Fully externally funded |  |  |
| Partially externally funded |  |  |
| Fully internally funded (e.g. core organization budget) |  |  |
| Partially internally funded |  |  |
| Participants pay for PARS |  |  |
| Other (define) |  |  |
| **Level 2 PARS Characteristics** |  |  |
| **2a Staff structure** | Tick all that apply | Define |
| Contracted staff |  |  |
| Self-employed |  |  |
| Volunteers |  |  |
| Other (define) |  |  |
| **2b Staff qualifications** | Tick all that apply | Define exact qualifications held |
| PARS qualification |  |  |
| Condition specific qualification |  |  |
| Other (state) |  |  |
| **2c To the best of your knowledge, is the scheme based on one or more behaviour change theories?** |  | Please state if you know what theory your scheme is based on |
| Yes |  |  |
| No |  |  |
| **2d To the best of your knowledge, does the scheme use one or more behaviour change techniques?** |  | Please state if you know what techniques your scheme uses |
| Yes |  |  |
| No |  |  |
| **2e Referral Source** | Tick all that apply | No. of referrals per year |
| Primary care |  |  |
| Secondary care |  |  |
| Tertiary care |  |  |
| Self-referral |  |  |
| Other (state) |  |  |
| **2f Referrers** | Tick all that apply | Additional comments |
| General practitioner |  |  |
| Practice nurse |  |  |
| Rehabilitation professional (state profession) |  |  |
| Self-referral |  |  |
| Social prescriber (e.g. link worker/health trainer) |  |  |
| **2g Referral process** | Tick all that apply | Additional comments |
| Email |  |  |
| Printed and mailed to participant |  |  |
| Printed and given to participant to take to PARS |  |  |
| Via online portal |  |  |
| Other (define) |  |  |
| **2h Scheme duration** | Tick one | State exact duration |
| Number of weeks client can attend scheme |  |  |
| Total number of sessions |  |  |
| No defined length (open-ended) |  | n/a |
| Other |  |  |
| **2i Session frequency** | State |  |
| Number of sessions per participant, per week |  |  |
| **2j Session length** | State |  |
| Define session length |  |  |
| **2k Session time** | Tick all that apply | Define time span of available sessions (e.g. 10.00-12.00pm) |
| Morning |  |  |
| Afternoon |  |  |
| Evening |  |  |
| Weekday |  |  |
| Weekend |  |  |
| **2l Physical activity session type** | Tick all that apply | Further define session type (e.g. PARS supervised circuit session or independent walking football option) |
| PARS-supervised group-based sessions |  |  |
| PARS-supervised individual sessions |  |  |
| Independent PA following assessment |  |  |
| Generic PARS-supervised sessions for all conditions |  |  |
| Condition specific PARS-supervised sessions |  |  |
| Independent PA choices without assessment |  |  |
| PA education sessions |  |  |
| Technology-based support *(e.g. mHealth app or web-based)* |  |  |
| Other (define) |  |  |
| **2m Exit routes** | Tick all that apply | Give details of exit routes activities |
| Formal exit route (defined sessions for completers) |  |  |
| Signposting to other activities |  |  |
| Open-ended (no exit route required) |  |  |
| None (state why no exit route provided) |  |  |
| **2n Action in case of non-attendance**  *Is there a standardised procedure for non-attendance?* | Tick all that apply | Specify time points, number of attempts to contact and by whom |
| **Yes** |  |  |
| Participant contacted by letter |  |  |
| Participant contacted by technology-based support |  |  |
| Participant contacted by telephone |  |  |
| Participant contacted by text |  |  |
| Participant not contacted |  |  |
| Other (define) |  |  |
| **No** |  |  |
| **2o Baseline assessment** | Tick one | State when this occurs (e.g. prior to first PA session or at first PA session) |
| Yes |  |  |
| No |  |  |
| **2p Exit assessment** | Tick one | State when this occurs (e.g. after 12 weeks, or after 24 sessions) |
| Yes |  |  |
| No |  |  |
| **2q Feedback provided to referrer** | Tick all that apply | State what is included and how feedback is provided (e.g. attendance and via email). |
| **Yes** (state what is included) |  |  |
| How is feedback provided? (state) |  |  |
| **No** |  |  |
| **2r Exclusion criteria** | Tick all that apply | State specific exclusion criteria |
| **Yes** |  |  |
| **No** |  |  |
| **Level 3 Participant Measures** |  |  |
| **3a Demographics** |  |  |
| ***3a.1 Sex*** | Tick if recorded | Define |
| State categories |  |  |
| ***3a.2 Age at the point of referral*** | Tick if recorded | Define |
| Individual age recorded |  |  |
| Minimum age |  |  |
| Maximum age |  |  |
| ***3a.3*** ***Socio-economic status*** | Tick if recorded | Define |
| Postcode/zipcode recorded |  |  |
| ***3a.4 Ethnicity*** | Tick if recorded | Define |
| State ethnic categories |  |  |
| ***3a.5 Employment status*** | Tick if recorded | Define |
| State employment categories |  |  |
| ***3a.6 Education status*** | Tick if recorded | Define |
| State education categories |  |  |
| ***3a.7 Other demographic measure*** | Tick if recorded | Define |
| State what other measures and how they are defined |  |  |
| **3b Monitoring and evaluation** |  |  |
| ***3b.1 Number of referrals*** | Tick if recorded | Additional comments |
| Number of referrals received per annum |  |  |
| ***3b.2 Uptake, attendance and adherence***  ***(please identify the definitions used)*** | Tick if recorded | Define measures |
| Uptake of intervention *(number of referrals who attend baseline assessment)* |  |  |
| Uptake of PA *(number of referrals that attend at least one PA session)* |  |  |
| Adherence to intervention *(number of referrals that attend exit assessment)* |  |  |
| Adherence to PA *(number of referrals that attend an agreed number of sessions e.g. 60% of programme contact time)* |  |  |
| Attendance *(number of attendances in a defined period)* |  |  |
| **3c Measures of change** | Tick if recorded | Define time points (e.g. baseline and week 24). |
| Change in PA behaviour (define measure) |  |  |
| Change in wellbeing (define measure) |  |  |
| Change in physiological measures (e.g. BP, weight, % weight change, BMI) |  |  |
| Other (define) |  |  |
